# Genotyping‐by‐sequencing analysis of *Orobanche crenata* populations in Algeria reveals genetic differentiation

**DOI:** 10.1002/ece3.8750

**Published:** 2022-03-24

**Authors:** Farah Bendaoud, Gunjune Kim, Hailey Larose, James H. Westwood, Nadjia Zermane, David C. Haak

**Affiliations:** ^1^ Department of Botany Ecole Nationale Supérieure Agronomique, ENSA Algiers Algeria; ^2^ Department of Plant Pathology, Physiology and Weed Science Virginia Tech Blacksburg Virginia USA; ^3^ School of Plant and Environmental Sciences Virginia Tech Blacksburg Virginia USA; ^4^ Faculty of Sciences University of Algiers Algiers Algeria

**Keywords:** Algeria, GBS, genetic diversity, genotyping by sequencing, *Orobanche crenata*, population structure

## Abstract

Crenate broomrape (*Orobanche crenata* Forsk.) is a serious long‐standing parasitic weed problem in Algeria, mainly affecting legumes but also vegetable crops. Unresolved questions for parasitic weeds revolve around the extent to which these plants undergo local adaptation, especially with respect to host specialization, which would be expected to be a strong selective factor for obligate parasitic plants. In the present study, the genotyping‐by‐sequencing (GBS) approach was used to analyze genetic diversity and population structure of 10 Northern Algerian *O*. *crenata* populations with different geographical origins and host species (faba bean, pea, chickpea, carrot, and tomato). In total, 8004 high‐quality single‐nucleotide polymorphisms (5% missingness) were obtained and used across the study. Genetic diversity and relationships of 95 individuals from 10 populations were studied using model‐based ancestry analysis, principal components analysis, discriminant analysis of principal components, and phylogeny approaches. The genetic differentiation (*F*_ST_) between pairs of populations was lower between adjacent populations and higher between geographically separated ones, but no support was found for isolation by distance. Further analyses identified four genetic clusters and revealed evidence of structuring among populations and, although confounded with location, among hosts. In the clearest example, *O*. *crenata* growing on pea had a SNP profile that was distinct from other host/location combinations. These results illustrate the importance and potential of GBS to reveal the dynamics of parasitic weed dispersal and population structure.

## Graphical abstract

Understanding the factors that shape obligate parasitic plant populations provides an important window on the evolutionary history of these unique plants. Using GBS‐derived markers across a number of *Orobanche crenata* populations we identified population genetic structure associated with A) geographic location and B) host plants. These data suggest that differentiation among these populations could result from the interaction of geographic separation and host specialization.

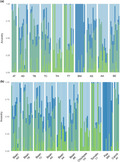

## 1 INTRODUCTION

*Orobanche crenata* (Forsk.), commonly called crenate broomrape, is a serious weed of many economically important crops (Parker, 2012). It is 1 of about 150 species in the genus *Orobanche* (Orobanchaceae) (Wolfe et al., 2005), which are notable for their parasitic mode of nutrition. Like some other members of this family, *O*. *crenata* lacks chlorophyll and photosynthetic capacity, so is completely dependent on autotrophic host plants for its nutritional requirements. The geographic distribution of the genus is mostly in the temperate and subtropical regions of the world, but centered in the Mediterranean area (Satovic et al., 2009; Zhang et al., 2014).

*Orobanche crenata* constitutes a major constraint to faba bean (*Vicia faba* L.) cultivation (Acharya, 2013; Pérez‐de‐Luque et al., 2010). However, this parasite also attacks crops such as lentil (*Lens culinaris* Medik.), pea (*Pisum sativum* L.), chickpea (*Cicer arietinum* L.), tomato (*Solanum lycopersicum* L.), lettuce (*Lactuca sativa* L.), and carrot (*Daucus carota* L.) (Aksoy et al., 2016; Renna et al., 2015; Román, Hernández, et al., 2007). Control of *O*. *crenata* is difficult due to its ability to produce high numbers of tiny seeds (up to 500,000 per plant) that can lie dormant in the soil for up to 20 years in the absence of a host (Habimana et al., 2014; Yahia et al., 2015). The parasite thus persists through seasons when hosts are not present, only to reappear when compatible host crops are replanted. Furthermore, the parasite is largely hidden below ground as the seedlings attach to host roots and inflict much of their damage to the host before the parasite floral shoot emerges from the soil. Several methods have been advocated for control of this weed, ranging from hand pulling, herbicides, biological control, delayed crop sowing, and crop rotation, but each of these suffer disadvantages due to economic constraints or limited effectiveness (Eizenberg et al., 2013; Kannan & Zwanenburg, 2014; Sheoran et al., 2016).

In Algeria, *O*. *crenata* is the major *Orobanche* species and is a serious problem for legume crops, mainly faba bean, pea, and chickpea. This parasite has been reported in several regions of Algeria, with high levels of infestation leading to the complete destruction of affected crops in some localities which force farmers to give up growing legume crops (Labrada, 2008). *Orobanche crenata* is a long‐standing agricultural problem in Algeria. The oldest herbarium specimens date to 1908 and were collected from legume crops in the region of El‐Harrach (previously called "Maison Carrée" during the French colonial period). History tells us of the extent *Orobanche* damage at the beginning of the last century. In 1923, Ducellier wrote the following: “*Faba beans and peas cultivation is made impossible in certain localities of the Sahel of Algiers and of the plateau of ‘Maison carrée’*, *so much has become common there*, *in the last fifteen or twenty years*, *the crenate broomrape*.″ At that time, the same author estimated that in some localities 60% of the land had become unsuitable for the cultivation of pea and faba bean as a result of the damage caused by this broomrape, which could lead to the complete crop failure (Blanchard, 1952). More than 70 years after Ducellier's statements, the *Orobanche* problem continues to increase. The parasite not only was reported to be still widespread in the Sahel of Algiers on legumes (Zermane, 1998) but also was found in the "Ain Dem" region (at "Khemis Méliana" town, about 200 km west of Algiers) causing significant losses on the same crops (Mahmoudi, 1993).

**FIGURE 1:**
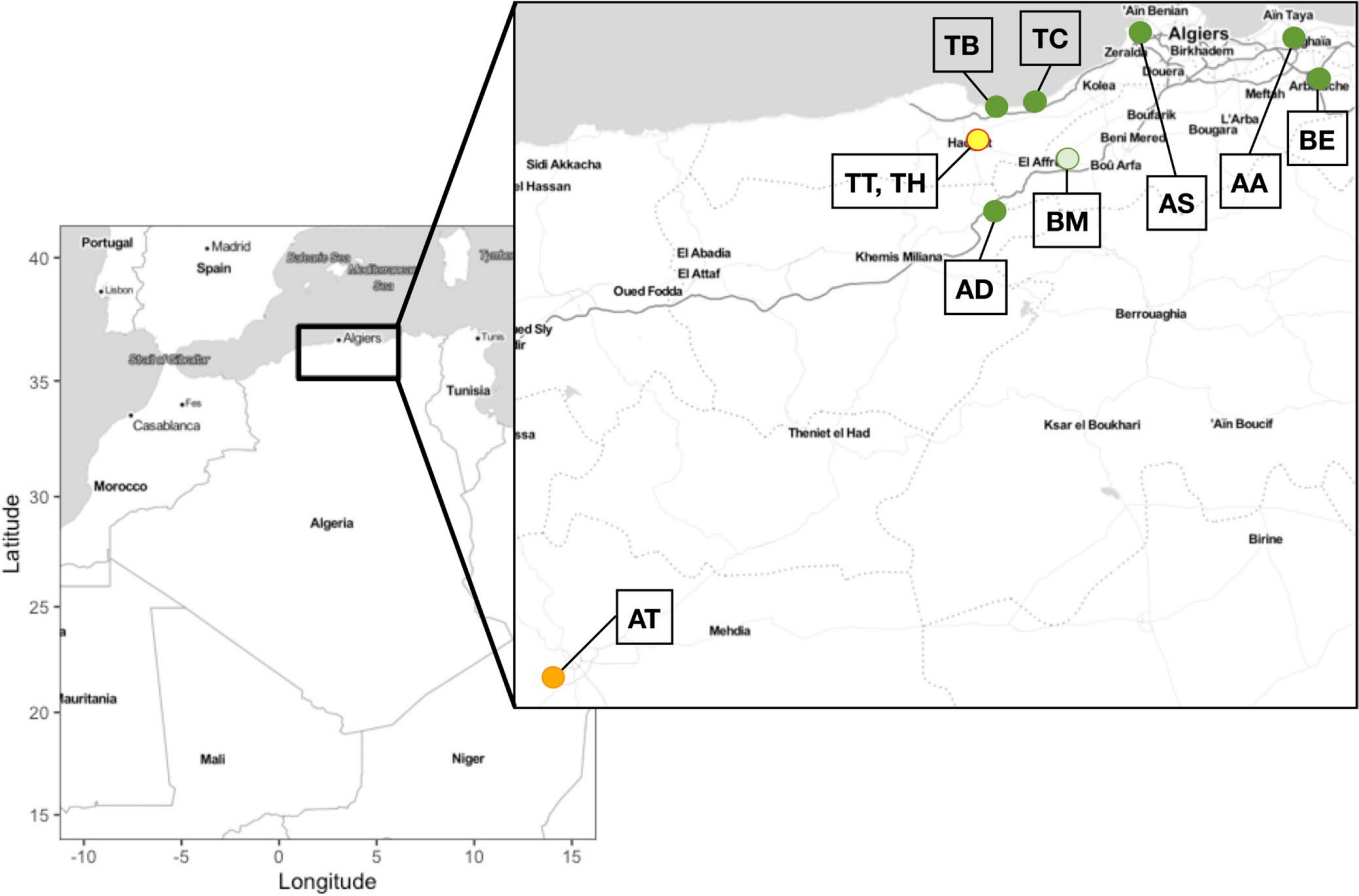
Sampling locations for 10 collections of *Orobanche crenata* from north central Algeria used in this study. Two letter designations indicate the collection and colors indicate the host crop, dark green = faba bean, light green = pea, yellow with red outline = chickpea and tomato, orange = carrot (described in Table 1)

A previous study was aimed to understand the genetic diversity of this species in Algeria using RFLP and RAPD markers (Aouali et al., 2007). This showed a proportional increase in genetic distance with geographical distance and suggested that the center of dissemination for this parasitic plant might be the region of “Mitidja,″ which is near the Ain Taya (Algiers) location used in the present study (Figure 1). Studies in other regions, employing ALFP and RAPD makers (Paran et al., 1997; Román et al., 2002) as well as microsatellites (SSR; Belay et al., 2020), have generally found high levels of among‐population individual variation and evidence of some degree of population structuring. In the case of comparisons across Spain and Israel (Román et al., 2002), in spite of evident gene flow, differentiation was found between countries and also among regions within, with populations in Israel showing greater differentiation than those found in Spain. Similarly, Belay et al. (2020) identified some population differentiation (two genetic clusters) among SSR markers in northern Ethiopia and gene flow among populations with little evidence of geographic separation.

In recent years, improved molecular techniques have been developed for genetic analysis of populations (Satovic et al., 2009). Advances in next‐generation sequencing technologies have enabled a revolution in genetic research through the ability to generate large numbers of single nucleotide polymorphisms (SNPs; Crossa et al., 2013). Genotyping by sequencing (GBS) is a high‐throughput genotyping platform that integrates SNP discovery and genotype calling into one step by reducing genome complexity via restriction enzymes (Elshire et al., 2011). It is an attractive technology for genomic selection by providing new cost‐effective opportunities for breeders because it generates large numbers of SNPs for exploring within‐species diversity, constructing haplotype maps, genome‐wide association studies, and genomic selection (Poland & Rife, 2012). The reduced representation of the genome and the barcoding of each individual enable multiple samples to be sequenced in one lane, leading to low‐cost genotyping of many individuals (Elshire et al., 2011).

Given the tremendous economic impact of *O*. *crenata*, the study of the genetic variation in this parasitic weed is important because it could lead to better understanding of *O*. *crenata* spread and adaptation. Furthermore, understanding how populations are genetically structured can provide some insight on how genetic variation in this species is shaped by evolution. In the present study, the GBS approach was used to identify and genotype SNPs in northern Algerian *O*. *crenata* populations that represent diversity in terms of geography and host species, with the aim to understand the population structure and geographical distribution.

## 2 MATERIAL AND METHODS

### 2.1 Plant material

A total of 100 emerged *O*. *crenata* shoots, comprising of 10 different plants from 9 populations and 5 different plants from 1 population, were collected from agricultural fields in Northern Algeria during spring of 2015. The details for each population, including collection site name, GPS coordinates, and host are displayed in Table 1 and Figure 1. The sampling was conducted with the objective of capturing the geographic range of *O*. *crenata* in Algeria, as well as host diversity, however, sampling was limited by available host crop. To this end, six populations were collected from faba bean hosts that represent the predominantly affected crop, and four populations were taken from other host species, carrot, chickpea, pea, and tomato.

**TABLE 1:** Collection locations and host information for the *Orobanche crenata* populations used in this study

Code	Region	Latitude/Longitude	Host
AA	Algiers (Ain Taya)	N 36°44′17.3″ E 003°18′20.4″	Faba bean
AD	Ain Defla	N 36°21'24.7″ E 002°28′58.8″	Faba bean
AS	Algiers (Staouali)	N 36°44203257.7″ E 002°52′58,1″	Faba bean
AT	Ain Timouchent	N 35° 19′22.8″ E 001° 16′17.0″	Carrot
BE	Boumerdes	N 36°38′58.3″ E 003°21′59,9″	Faba bean
BM	Blida (Mouzaia)	N 36°28′24.1″ E 002°40′43.4″	Pea
TB	Tipaza (Bouharone)	N 36°35′54.8″ E 002°35′25.5″	Faba bean
TC	Tipaza (Chenoua)	N 36°35′23.4″ E 002°29′18.0″	Faba bean
TH	Tipaza (Hadjout)	N 36°30′48.9″ E 002°26′03.5″	Chickpea
TT	Tipaza (Hadjout)	N 36°31′00.6″ E 002°26′02,7″	Tomato

### 2.2 Genotyping by sequencing

Genomic DNA was extracted from floral buds using Qiagen DNeasy Plant Mini kit (QIAGEN Strasse 1, 40724 Hilden, Germany) following the manufacture's instruction. Samples were sent to the Institute of Genomic Diversity at Cornell University for genotyping by sequencing, libraries were prepared as described in the protocol by Elshire et al. (2011). Briefly, the DNAs from 96 individuals were digested with EcoT22I 6‐base cutter (ATGCAT) to reduce the genome complexity. A 96‐plex GBS library comprising 95 DNA samples and a negative (no DNA) were prepared by ligating the digested DNA to unique barcode nucleotide adapters, followed by standard PCR. The resulting 96‐plex library was sequenced on a single lane of an Illumina HiSeq 1 × 100 bp.

### 2.3 Sequencing data analysis and SNP calling

Raw sequence data were processed using the Universal Network‐Enabled Analysis kit (UNEAK) pipeline implemented in the Iplant collaborative platform. This pipeline produced a hapmap file for downstream analysis. This file was used as input for SNP identification using the GBS pipeline implemented in TASSEL (Version: 3.0.166). Raw SNPs were filtered following the dDocent guidelines (Puritz et al., 2014). In short, using vcftools (Danecek et al., 2011) variants were filtered for depth >5, quality >Q30, and initially 50% missingness. This file was used to screen samples for high levels of missingness (all were <30%). The final SNP set was filtered for a maximum of 5% missing values and a minor allele frequency <0.05.

### 2.4 Population differentiation and genetic diversity

The TASSEL‐derived vcf was converted into formats compatible with downstream analyses using PGDSpider v2.0.9.0 (Lischer & Excoffier, 2012). Population summary statistics were generated using “basic.stats,” “fstat,” and “pairwise.WCfst” from the hierfstat v0.5‐7 package (Goudet, 2005) in the R v4.0.4 computing environment (R Core Team, 2013). Poppr v2.9.0 (Kamvar et al., 2014) was used to analyze genetic distances between populations and conduct AMAOVAs across hosts and populations. Nei's genetic distance was calculated across the full set of SNPs while “missingno″ was used to purge markers with missing data from the SNP dataset for use in AMOVAs, and to test isolation by distance. Isolation by distance was tested using “mantel.randtest″ from the adegenet v2.1.3 package (Jombart & Ahmed, 2011) on distance matricies generated using “dist.genepop″ (with Edwards’ distance) for SNPs and “dist″ on latitude and longitude (decimal degrees) for each population.

### 2.5 Population structure

The number of genetic clusters across populations were identified using maximum‐likelihood hierarchical clustering via ADMIXTURE v1.3.0 as well as principal components analysis, and discriminant analysis of principle components as implemented in poppr v2.9.0. ADMIXTURE was implemented with 15 iterations for each k from 1 to 10. Cross‐validation was used to identify the optimal k. Cross‐validation was also used to identify the optimal number of principal components via “xvalDapc″ in poppr, which were used to identify the number of clusters in the data via “find.clusters.″ All data were visualized using poppr or ggplot v3.3.3 (Wickham, 2016). All scripts necessary to reproduce these analyses can be found here: figshare link.

## 3 RESULTS

### 3.1 Variants generated by GBS

In total 95 plants were digested, sequenced, and genotyped using GBS, representing 10 locations (Table 1, Figure 1). After quality filtering, a total of 242,207,242 reads were obtained from sequencing on a single lane of Illumina HiSeq 100bp single‐end reads. The pipeline Universal Network‐Enabled Analysis kit (UNEAK: Citation) as implemented in Iplant was used to derive single nucleotide polymorphisms (SNP). Variant calling resulted in a total of 59,437 raw SNP variants. After filtering for a mean depth of 10, quality score of 30, minor allele frequency (MAF) of 0.05, and 5% missingness, 8004 bi‐allelic SNPs were retained. After removing SNPs with missing data, we retained 2935 high‐quality SNPs among the 95 individuals across the study.

### 3.2 Population differentiation

Population differentiation was estimated using Weir and Cockerham’s (1984) *F*_ST_ (Table 2). *F*_ST_ estimates indicate a degree of separation among populations where 0 indicates that populations are sharing genes to 1 indicating complete isolation. The overall population *F*_ST_ was estimated at 0.0522 indicating some degree of structure among populations, supported by an exact test using the G‐statistic (population *p* = .002 and population nested within host *p* = .01). Pairwise *F*_ST_ values ranged from a low of 0.0162 (adjacent populations TH and TT: Figure 1) to a high of 0.1193 between geographically separated populations (AT, BM: Figure 1). Interestingly, the most geographically separated populations, AT and AA, appeared less genetically differentiated with an *F*_ST_ of 0.0858. Comparing pairwise *F*_ST_ estimates across crop hosts also indicated genetic differentiation (Table 3). Here, the lowest, 0.0162, between tomato (TT) and chickpea (TH), and the highest, 0.1193, between carrot (AT) and pea (BM), are confounded by short geographic distance. In contrast, *F*_ST_ between *O*. *crenata* growing on pea (BM) and chickpea (TH) was the second highest at 0.1135. Within faba bean host samples (AA, AD, AS, BE, TB, and TC), the pairwise *F*_ST_ values ranged from 0.0216 to 0.0354, indicating that there was less genetic differentiation within faba bean hosts than across all samples.

**TABLE 2:** Pairwise *F*_ST_ values (Weir and Cockerham) among *O*. *crenata* by collection location

	AA (Bean)	AD (Bean)	AS (Bean)	AT (Carrot)	BE (Bean)	BM (Pea)	TB (Bean)	TC (Bean)	TH (Chickpea)
AD (Bean)	0.0354								
AS (Bean)	0.0342	0.0076							
AT (Carrot)	0.0858	0.0594	0.0531						
BE (Bean)	0.0406	0.0219	0.0151	0.0562					
BM (Pea)	0.0954	0.0716	0.0551	0.1193	0.0661				
TB (Bean)	0.0388	0.0120	0.0117	0.0571	0.0130	0.0704			
TC (Bean)	0.0533	0.0277	0.0289	0.0901	0.0365	0.0810	0.0253		
TH (Chickpea)	0.0423	0.0357	0.0410	0.0799	0.0455	0.1135	0.0431	0.0559	
TT (Tomato)	0.0496	0.0393	0.0343	0.0707	0.0426	0.0981	0.0375	0.0505	0.0162

**TABLE 3:** Pairwise *F*_ST_ values (Weir and Cockerham) among *O*. *crenata* by host

	Bean	Carrot	Chickpea	Pea
Carrot	0.0512			
Chickpea	0.0311	0.0799		
Pea	0.0544	0.1193	0.1135	
Tomato	0.0300	0.0707	0.0162	0.0981

**FIGURE 2:**
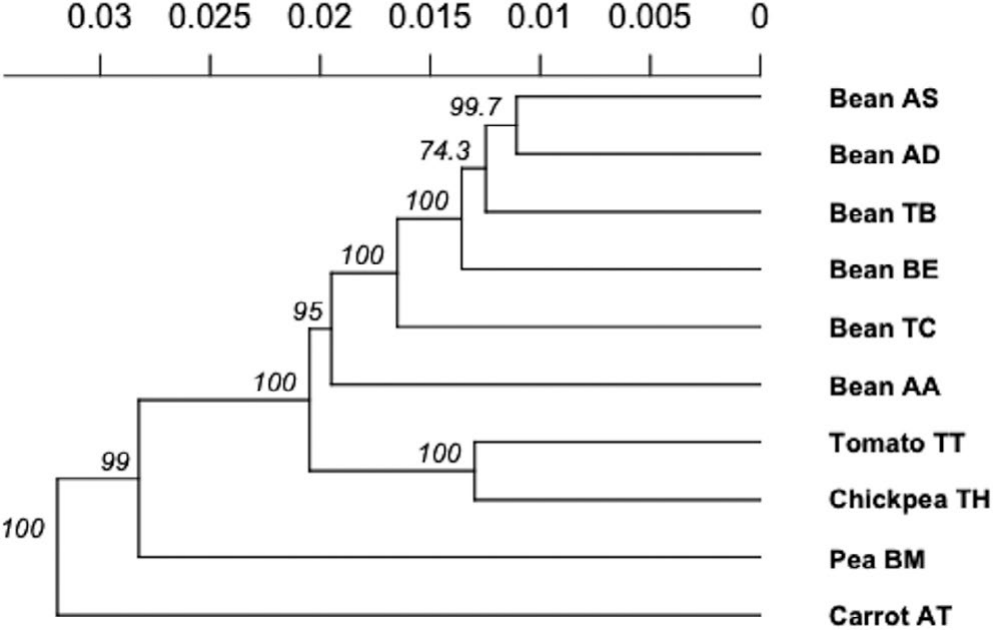
Neighbor joining (NJ) tree based on Nei's genetic distance. Node labels indicate bootstrap support across 1000 replicates

Nei's genetic distance (Nei, 1972) was calculated and used to construct a neighbor‐joining tree (Figure 2) which grouped samples by host plant with strong bootstrap support (99–100%). Intriguingly, while carrot (AT) is a geographic outlier, tomato (TT) and chickpea (TH), which were collected in adjacent fields, were split with 100% bootstrap support. Faba bean host samples were split into four groups with moderate bootstrap support (95%). Within this grouping, support for differentiation by location varied (74.3–100%).

### 3.3 Analysis of molecular variance and isolation by distance

A hierarchical AMOVA, as implemented in poppr with method = “ade4,″ was carried out using population within host (Table 4). Consistent with previous studies in *O*. *crenata* (Romàn et al., 2001), the majority of variation is found within individuals, suggesting very little population differentiation. However, there is some evidence for separation by host (phi = 0.031, *p* = .004) and population within host (phi = .028, *p* = .001). The observed increased individual variation among *O*. *crenata* populations results from a significant excess of heterozygosity (*F*_IS_ = −0.22, Bartlett's K‐squared = 1969, *df* = 1, *p*‐value < 2.2e‐16).

**TABLE 4:** Analysis of molecular variance (AMOVA) summary of the genetic variation in *O*. *crenata* by location nested within host

	*df*	SS	MS	Sigma	*p*‐value	Phi
Between hosts	4	8134.92	2033.73	31.93	.004	0.031
Between populations within hosts	5	10818.89	2163.78	28.59	.001	0.028
Between individuals within populations	85	57168.10	672.57	−303.34	1.000	−0.310
Within individuals	95	121529.00	1279.25	1279.25	1.000	−0.234
Total	189	193053.91	1021.45	1036.43		

Abbreviations: *df*, degrees of freedom; MS, mean squares; phi, degree of population differentiation; SS, sums of squares.

**FIGURE 3:**
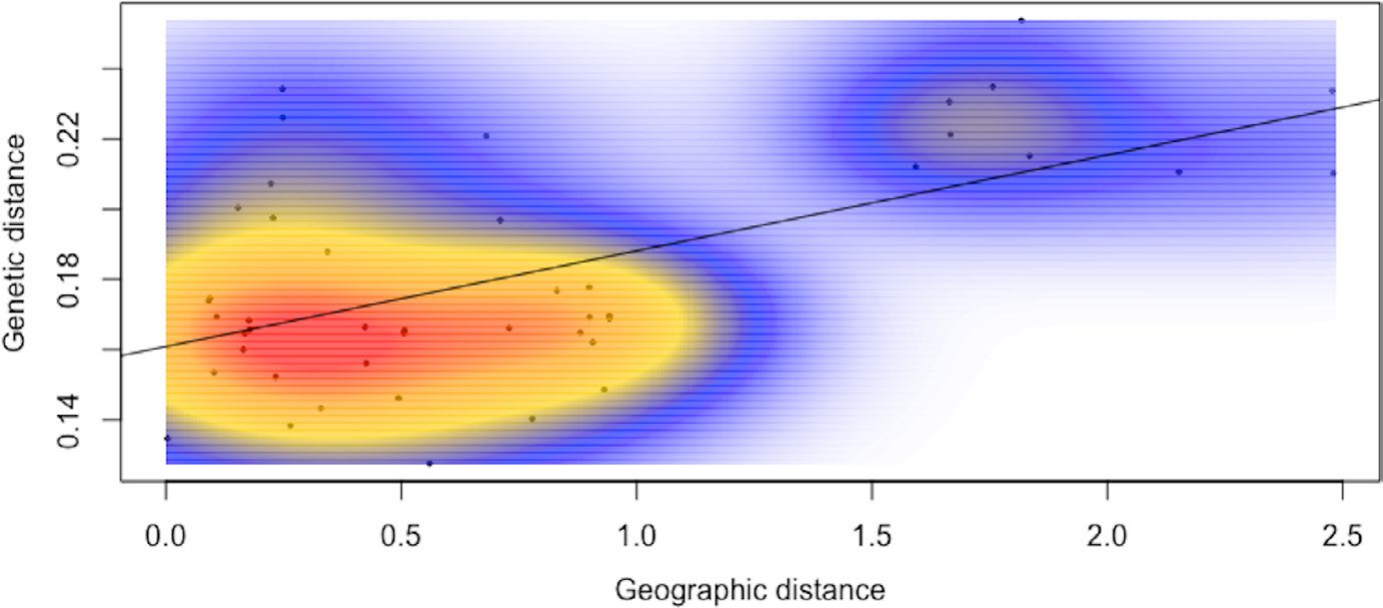
A plot of geographic distance by genetic distance (Edwards' distance) identifies a discontinuity between groups. This patch represents a geographically distant population (AT) and drives the linear relationship (dashed line, R2 = 0.28, *p* <<.01). Isolation by distance for all populations is not supported by a Mantel test (R = 0.55, *p* = .10)

To test the role of isolation by distance (IBD), a Mantel test was conducted using Edward's genetic distance and a geographic distance matrix (latitude, longitude). Overall, we find no support for IBD (*R* = 0.55, *p* = .10) across populations. We do find, however, two distinct patches in the kernel density estimates for IBD (Figure 3). This patchiness appears to be driven by the outlying population, AT, which exhibits moderate genetic differentiation, with mean *F*_ST_ = 0.075 (Table 2) and geographic distance, mean = 1.94. This outlying patch drives a significant linear relationship between genetic distance and geographic distance (dashed line Figure 3, *R* ^2^ = 0.283, *p* = .0001).

### 3.4 Population structure

**FIGURE 4:**
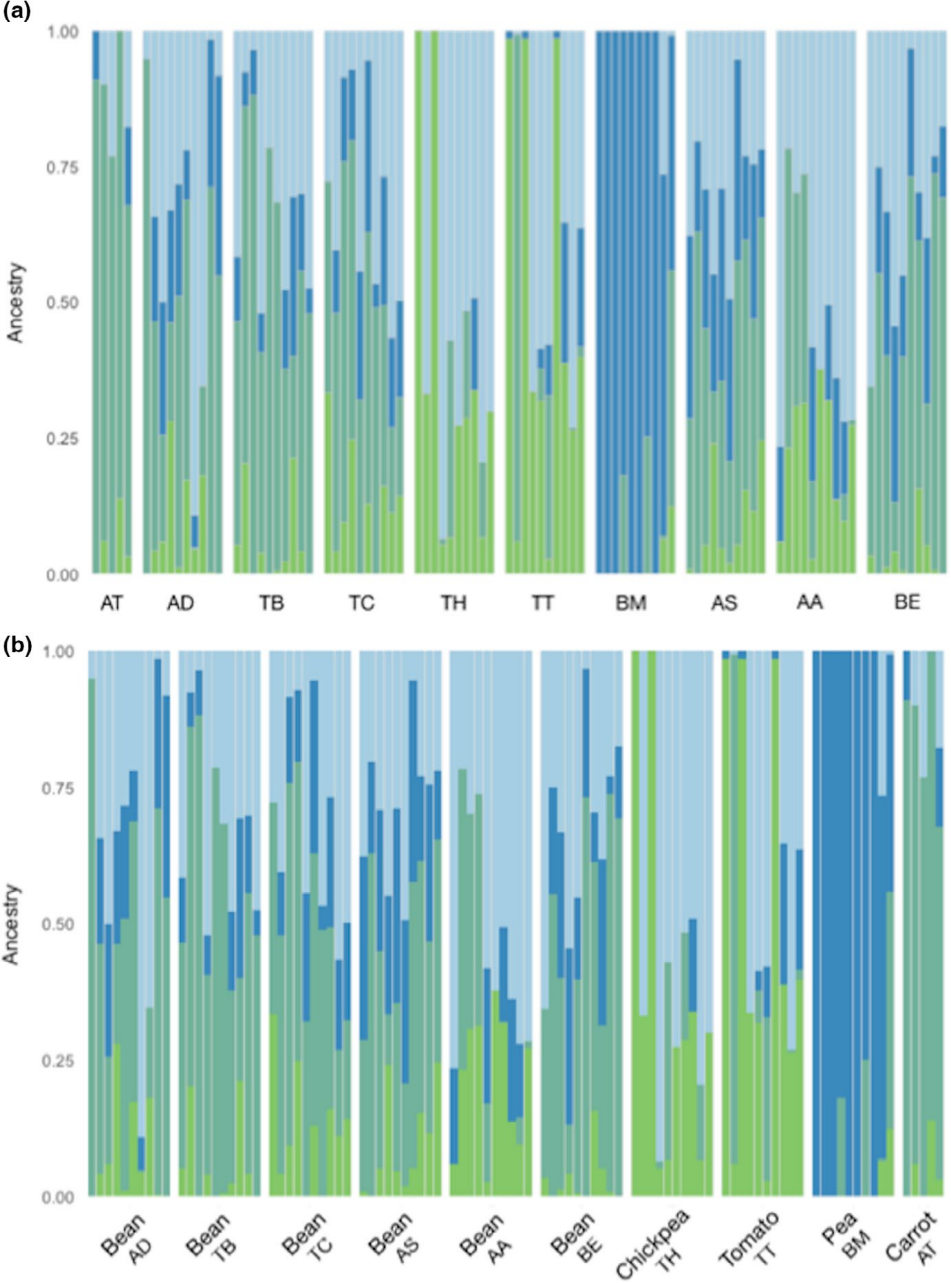
Population structure of Orobanche crenata based on ancestral clusters from Admixture arranged by (a) geographic location and (b) host crop. The number of clusters *K* = 4 was selected via cross validation from *k* = 1‐10 Admixture

The analysis of population structure was conducted using two different clustering approaches, model‐based maximum likelihood hierarchical clustering via ADMIXTURE (Alexander & Lange, 2011) and individual‐based principal component analysis (PCA) on genetic distances. Cross‐validation as implemented in ADMIXTURE across 15 replicates identified *K* = 4 as the optimal number of genetic clusters in the data (Figure S1). Visualization of the coefficients of ancestry for each individual is arrayed by geographic location from southwest to northeast (in northern Algeria, Figure 1) in Figure 4a. Figure 4b shows the admixture among populations as arranged by hosts. As with genetic distance estimates, ADMIXTURE reveals evidence of structuring among populations and hosts. In this case, patterns of structuring among hosts are more evident than strictly along a geographic gradient. For example, faba bean hosts have more similar patterns of shared ancestry than that shared between faba bean and chickpea or tomato, despite covering a broad range of geographical space. In contrast, populations AT and BM are spatially and genetically isolated. These data are consistent with the AMOVA results supporting a role for an interaction between host and location partitioning genetic variance among populations (Table 4).

**FIGURE 5:**
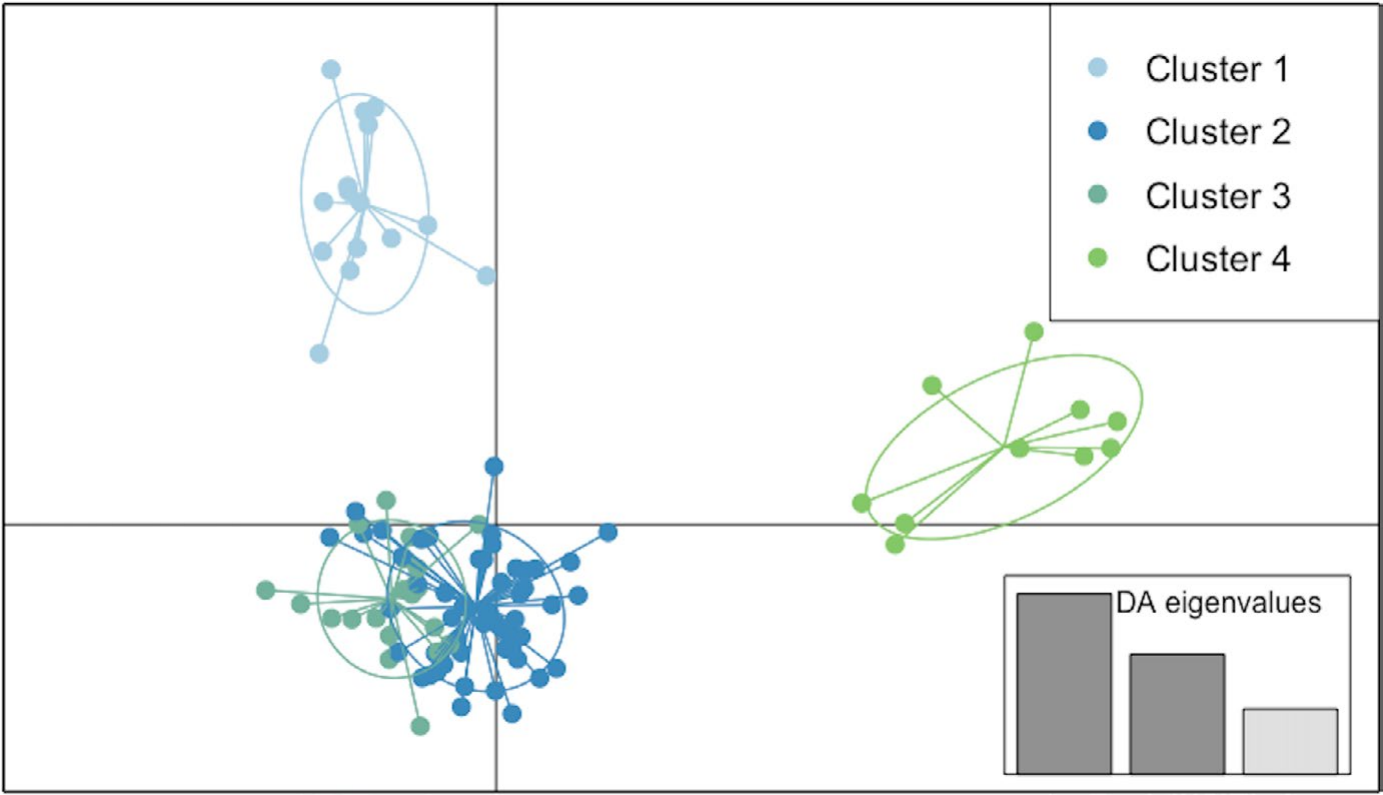
Clustering revealed 4 distinct groups that encompassed populations and hosts. Scatterplots from discriminant analysis of principle components (DAPC), where ellipses indicate the variance spanned by 95% of the data

**FIGURE 6:**
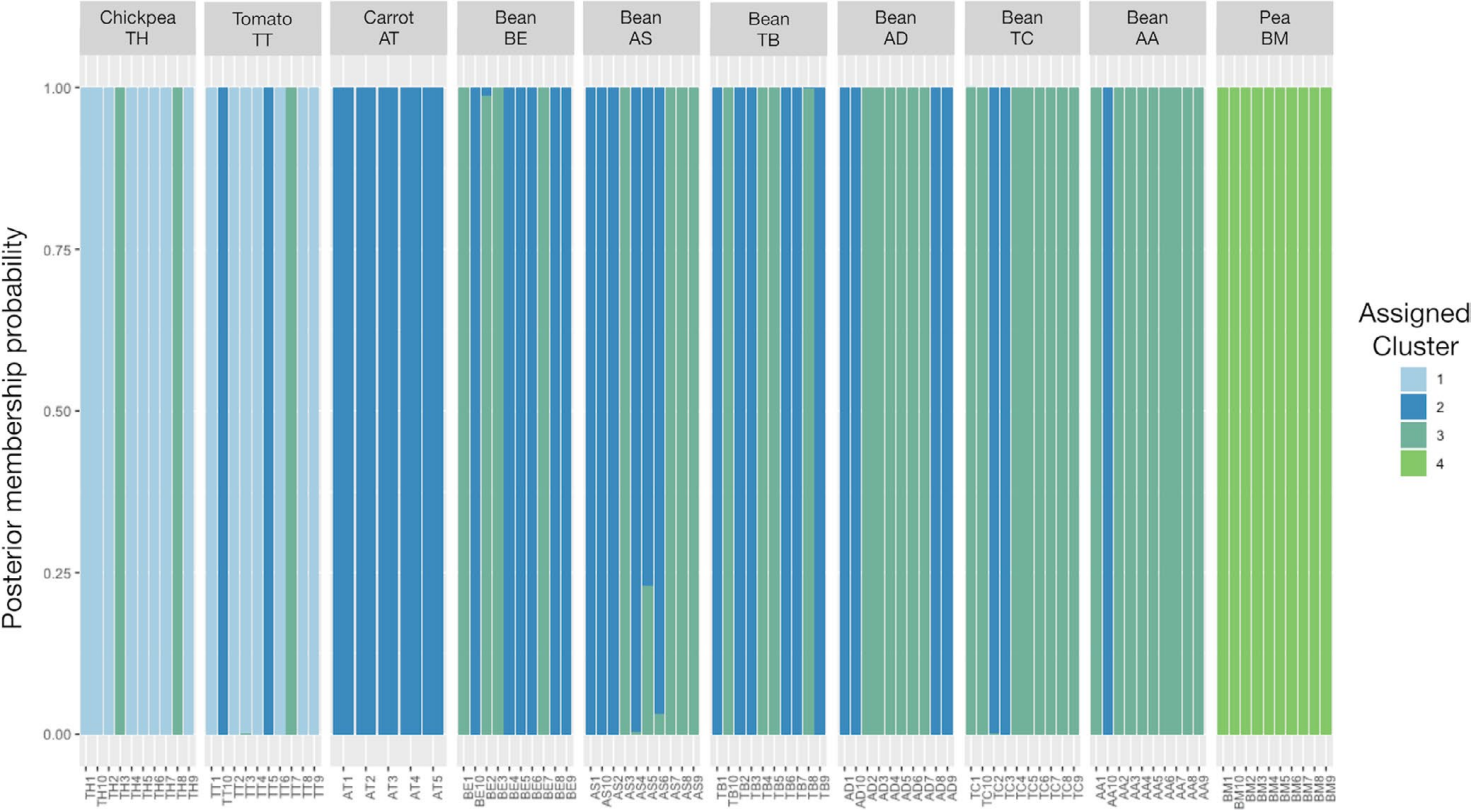
Assignment plot showing the individual posterior membership probabilities for each DAPC identified cluster grouped by collection location

The first 4 components from a PCA of genetic distance explained 66.16% of the total variation in the data. To further examine the relationship between samples within this PCA, we conducted a discriminant analysis of principle components (DAPC). Discriminant analysis of principle components identified four genetic clusters in the data across three axes (Figure 5). Cluster assignments supported both the model‐based and AMOVA identified genetic differentiation by host/population (Figure 6). Posterior assignment supports admixture within chickpea, tomato, and faba bean hosts but not within carrot or pea. Chickpea and tomato samples predominate cluster 1. Pea samples are all placed in cluster 4 while carrot samples are all placed in cluster 2. Faba bean samples have significant overlap between cluster 3 (*N* = 35) and cluster 2 (*N* = 22) with two samples having shared membership.

## 4 DISCUSSION

Despite the interest devoted to *Orobanche* spp. given their tremendous economic impact, knowledge of their genetic variability is still limited. Genetic diversity analysis is of great importance as it will facilitate understanding the genetic structure of parasitic weed populations. This knowledge will provide insights into parasite dispersal, host specialization, development of new races, and in establishing diverse collections of parasite races for use in crop breeding programs (Román, Hernández, et al., 2007; Román, Satovic, et al., 2007; Román et al., 2002; Vaz Patto et al., 2008). However, accurate genetic diversity studies require powerful and reliable genetic tools, such as molecular markers.

Throughout the last two decades, several studies attempted to elucidate patterns of genetic variation in *Orobanche* spp. using different molecular markers such as RFLP (Vaz Patto et al., 2008, on *O*. *foetida*), RAPD (Bouhadida et al., 2015; Román, Satovic, et al., 2007; Román, Hernández, et al., 2007, on *O*. *foetida*), ISSR (Román et al., 2002, on *O*. *crenata*), SSR (Belay et al., 2020, on *O*. *crenata*; Pineda‐Martos et al., 2014, on *O*. *cumana*), SSR‐SNP (Calderón‐González et al., 2019, on *O*. *cumana*), and SRAP (Ennami, Briache, Mbasani Mansi, et al., 2017, on *O*. *crenata*). In recent years, genotyping by sequencing (GBS) has become an ideal tool for plant breeding and plant genetics studies (Chung et al., 2017). To the best of our knowledge, there are no published studies on genetic variability in *Orobanche* spp. using GBS. Our work is therefore the first to use this approach for broomrape, generating 2935 high‐quality SNP markers that provided substantially higher resolution relative to earlier approaches. In the present study, the genetic diversity and population structure of 10 *O*. *crenata* populations originating from different locations and crop hosts in Algeria were analyzed by GBS‐SNPs.

### 4.1 Genetic differentiation of populations

In our study, the observed genetic differentiation (*F*_ST_) between pairs of *O*. *crenata* populations by collection location varied from 0.0076 to 0.1193 (Table 2) and was lower between adjacent populations and higher between geographically separated ones. Low values indicate a lower level of genetic differentiation, consistent with gene flow between populations. In this study, populations with low *F*_ST_ are geographically close and are located in five contiguous districts (Algiers, Tipaza, Ain Defla, Blida, and Boumerdes). This region is known for intensive vegetable cultivation where exchange and/or sharing and trading of agricultural material (crop seeds and seedlings, manure, machinery, etc.) are common practices (Bessaoud et al., 2019). These practices may have supported seed migrations and resulted in apparent gene flow among populations. Results from the AMOVA analysis (Table 4) also support the possibility of high rates of gene flow between locations since the majority of total variation (63%) was found within individuals and low average *F*_ST_ values between populations, which suggests very little population differentiation. These populations are therefore genetically close and likely evolved from the same source.

Previous studies in *O*. *crenata* populations from Morocco (Ennami, Briache, Mbasani Mansi, et al., 2017), Spain (Román et al., 2001, 2002), and Egypt (Abdalla et al., 2016) also reported a clear genetic variation at the intra‐population level and only little differentiation among populations. According to Musselman (1986) and Romàn et al. (2002), these results are expected considering the predominantly allogamous behavior of *O*. *crenata* and the extremely efficient dispersal of its seeds. Conversely, high *F*_ST_ values suggest reduced gene flow between populations leading to population differentiation. In our study, the highest *F*_ST_ values (0.0858–0.1193) were recorded for the most geographically distant populations, in particular that of Ain Temouchent (AT), which is the farthest collection location. Genetic differentiation among populations is expected to increase with increased geographic distance (Slatkin, 1993). Analysis of the pairwise genetic differentiation in our study provides some evidence that genetic distance between populations increased proportionally with geographic distance. This was also the conclusion of Aouali et al. (2007) regarding *O*. *crenata* populations from the plain of Mitidja in northern Algeria. However, a Mantel test revealed no support for isolation by distance (IBD) across these populations overall. Nonetheless, the Ain Temouchent population (AT) remained an outlier driving the linear relationship with geographic distance (Figure 3). In contrast, overall, estimates of *F*_ST_ are consistent with other studies of parasitic weeds and suggest that host and geography are important factors shaping genetic differentiation in *O*. *crenata* (Román, Hernández, et al., 2007; Román et al., 2002; Stojanova et al., 2019).

Results from prior research support the observation that inter‐population differentiation is likely to be detected between distant countries rather than within countries (Satovic et al., 2009). In explaining what could be responsible for this trend, Romàn et al. (2001, 2002) suggested that geographic distance provides a substantial barrier to gene flow as long as there is no commercial exchange of host seeds between the regions; whereas within a country migration forces between populations are continuous and strongly favored by an efficient dispersal of the parasite seeds via humans, machinery, animals, or wind, as well as on host seeds.

### 4.2 Population structure and host differentiation

Population differentiation by geographic distance was more evident across crop hosts and was lowest between tomato and chickpea in adjacent populations (TT& TH, *F*_ST_ = 0.0162) and highest between carrot and pea in distant populations (AT& BM, *F*_ST_ = 0.1193). These data are consistent with the AMOVA results (Table 4), supporting a role for an interaction between host and location partitioning genetic variance among populations.

Clustering analyses supported the existence of four clusters based on genetic differentiation by host/population. Cluster 1 contained predominantly populations from “Tipaza‐Hadjout″ (90 m altitude/635 mm annual rainfall/18.5°C annual average temperature) harvested on chickpea (TH) and tomato (TT) samples. Cluster 2 is made exclusively of carrot samples all from the population (AT) of “Ain Temouchent″ (235 m alt. / 485 mm ann. rainfall/17.4°C ann. average temp.). Cluster 4 grouped pea samples, all from the population (BM) from “Blida‐Mouzaia″ (120 m alt./684 mm ann. rainfall/18.1°C ann. average temp.). Cluster 3 and cluster 4 shared populations harvested on faba bean (AA, AD, AS, BE, TB, and TC), almost all from the coastline (except AD: 1 to 41m alt. / 619 to 739 mm ann. rainfall/17.7 to 18.7°C ann. average temp.).

While overall populations maintain close genetic relatedness, presumably driven by gene flow, this number of clusters suggest that the sampled *O*. *crenata* populations are differentiated across this range. This is consistent with multiple possible evolutionary processes including drift associated with population isolation but also the possibility that host specialization and adaptation to local ecological conditions are driving population differences. For example, the most geographically separated populations AT and AA (432 km straight line distance), expected to have the highest *F*_ST_ value, appeared less genetically differentiated with an *F*_ST_ of 0.0858. This outcome is consistent with the hypothesis that both populations could have evolved from the same origin and that AT became differentiated from other populations (although the present study could not discern mechanism). This is also consistent with the possibility of spread of *O*. *crenata* seeds from northeast to southwest (within northern Algeria). This direction seems most plausible since the oldest infestations were reported in the central part of the Algiers coastline (Sahel of Algiers), which is consistent with the study of Aouali et al. (2007) that suggested the center of dissemination of *O*. *crenata* might be the region of “Mitidja.”

In addition, from data in Table 2, populations AT and BM appeared to share fewer genes with all other populations as they present in general the highest pairwise *F*_ST_ values. It is worth noting that geographically, the population in Blideen Atlas (BM) contrasts with the majority of the populations located on the coastal plains. This geographic structuring could have eventually allowed the isolation of this population (Welsh & Mohamed, 2011). It is possible that genetic isolation at Blideen Atlas also could have resulted from local ecological and geographic features, host specialization, the interaction of these, or selection pressure for agronomic traits. Importantly, the present study cannot distinguish this outcome from other processes (e.g., genetic drift) that could have led to this differentiation. More populations across hosts and more locations are needed to better understand how and when host genetic differentiation occurs.

Several authors have investigated host differentiation among parasitic weeds, with sometimes contrasting results. Aouali (2005) could not find evidence of host differentiation among eight *O*. *crenata* populations parasitizing faba bean, chickpea, pea, and carrot in the Mitidja plain in Algeria using RAPD and RFLP markers. Similarly, Ennami, Briache, Gaboun, et al. (2017) b) did not detect host specialization among *O*. *crenata* accessions from three regions in Morocco collected from faba bean and lentil hosts using RAPD markers. Conversely, evidence of host differentiation has been found in other parasitic weeds, such as *O*. *foetida* (Román, Satovic, et al., 2007; Román, Hernández, et al., 2007; Vaz Patto et al., 2008, Thorogood et al., 2008; but *not* Boukteb et al., 2021), *Striga hermonthica* (Unachukwu et al., 2017), and *Phelipanche ramosa* (Stojanova et al., 2019). These differences may be due to specific methods/sampling design or may arise from the type of genetic marker selected as single locus co‐dominant markers are more efficacious for population biology insights (Sunnucks, 2000).

The present study provides relevant population genetic information that may benefit future breeding programs and management practices aimed at bolstering resistance against this parasitic weed. Other aspects that are worth further investigation may include cross‐infestation experiments to ascertain host preferences and specialization (Román, 2013; Stojanova et al., 2019). Also, it would be interesting to study genetic interactions between wild and weedy forms of *O*. *crenata* (Satovic et al., 2009). In Algeria, the host range of *O*. *crenata* includes both cultivated and wild plant species belonging to at least eight families. Host crops include: *Carthamus tinctorius*, *Cicer arietinum*, *Daucus carota*, *Lactuca sativa*, *Lathyrus sativus*, *L*. *ochrus*, *Lens esculenta*, *Lupinus* sp., *Pisum sativum*, *Solanum lycopersicum*, *Vicia faba*, and *V*. *sativa*. Wild hosts include: *Dipsacus* sp., *Geranium* sp., *Lathyrus odoratus*, *Medicago hispida*, *Pichris echioides*, *Plantago lanceolata*, and *Trapaeolum majus*. While not yet investigated, host relationship studies between wild and weedy *O*. *crenata* populations in Algeria would provide useful insights since wild vegetation may act as a reservoir of genetic diversity for overcoming genetic resistance mechanisms in host crops (Pineda‐Martos et al., 2014).

## 5 CONCLUSIONS

In this study we explored the genetic diversity, population structure, and potential host differentiation of 95 individuals from 10 populations of *O*. *crenata* collected from different locations and crop hosts in Algeria using GBS‐derived markers. A set of 8004 high‐quality SNPs (5% missingness, 2935 with 0% missingness) was generated for the genetic diversity analyses. The study revealed low‐to‐moderate genetic differentiation between close and geographically separated populations, respectively. Population differentiation by geographic distance was more evident when coupled with crop hosts as supported by phylogenetic and clustering analyses. Four genetic pools were differentiated, clustered according to crop hosts, although this was confounded by population separation. AT and BM (Blida‐Mouzaia) populations were shown to be spatially and genetically isolated. This study identifies genetic differentiation among *O*. *crenata* populations in northern Algeria. Future studies are needed to identify the evolutionary processes shaping this differentiation.

## CONFLICT OF INTEREST

All authors declare no conflicts of interest.

## AUTHOR CONTRIBUTIONS

**Farah Bendaoud:** Investigation (equal); Methodology (equal); Writing – original draft (equal); Writing – review & editing (equal). **Gunjune Kim:** Investigation (supporting); Methodology (supporting); Software (supporting); Supervision (supporting); Validation (supporting); Writing – original draft (supporting); Writing – review & editing (supporting). **Hailey Larose:** Investigation (supporting); Methodology (supporting); Project administration (supporting); Writing – review & editing (supporting). **James H. Westwood:** Conceptualization (equal); Funding acquisition (equal); Investigation (supporting); Project administration (equal); Resources (equal); Supervision (equal); Writing – original draft (equal); Writing – review & editing (equal). **Nadjia Zermane:** Conceptualization (equal); Funding acquisition (lead); Investigation (equal); Project administration (equal); Resources (equal); Writing – original draft (equal); Writing – review & editing (equal). **David C. Haak:** Data curation (lead); Formal analysis (lead); Investigation (equal); Methodology (equal); Project administration (equal); Resources (equal); Supervision (lead); Validation (equal); Visualization (lead); Writing – original draft (lead); Writing – review & editing (lead).

### OPEN RESEARCH BADGES

This article has been awarded Open Materials, Open Data Badges. All materials and data are publicly accessible via the Open Science Framework at https://doi.org/10.7294/14838213.

## Supporting information

Figure S1

## Data Availability

All GBS sequence data are available under BioProject PRNJ742536 (http://www.ncbi.nlm.nih.gov/bioproject/742536). A sample manifest with the barcodes and sample ids, all R scripts for population analyses, and associated data files can be found at VTechData: https://doi.org/10.7294/14838213.
