# Supplementary material for: Genotyping‐by‐sequencing analysis of Orobanche crenata populations in Algeria reveals genetic differentiation
Source: Ecol Evol. 2022 Mar 24;12(3):e8750. doi: 10.1002/ece3.8750 (PMC8948082; doi:10.1002/ece3.8750)

**Supplemental Information for:**

GBS analysis of *Orobanche crenata* populations in Algeria supports local adaptation and host-specialization

Farah Bendaoud^1^, Gunjune Kim^2^, Hailey Larose^2^, James H. Westwood^2,3^, Nadjia Zermane^4^* and David C. Haak^2,3^*

Figure S1. Cross-validation plot for values of K from Admixture.


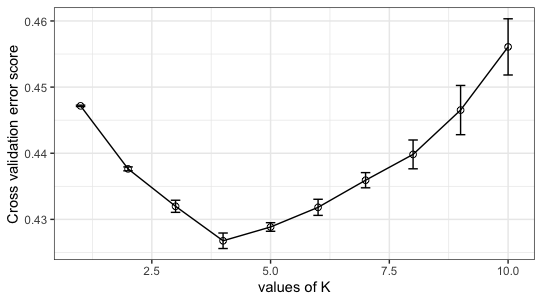

Supplement: Supplementary file 1 — Figure S1 [file ECE3-12-e8750-s001.docx]
